# Development of a Novel Technique to Dissect the Mesentery That Preserves Mesenteric Continuity and Enables Characterization of the *ex vivo* Mesentery

**DOI:** 10.3389/fsurg.2019.00080

**Published:** 2020-01-23

**Authors:** Ashutosh Kumar, Muneeb A. Faiq, Hare Krishna, Vijay Kishan, Gladwin V. Raj, John Calvin Coffey, Tony George Jacob

**Affiliations:** ^1^Department of Anatomy, All India Institute of Medical Sciences (AIIMS), Patna, India; ^2^Langone Health Center, New York University School of Medicine, New York, NY, United States; ^3^Department of Anatomy, All India Institute of Medical Sciences (AIIMS), Jodhpur, India; ^4^Mahatma Gandhi Medical College and Research Institute (MGMCRI), Puducherry, India; ^5^Department of Anatomy, Jawaharlal Institute of Postgraduate Medical Education and Research (JIPMER), Puducherry, India; ^6^Graduate Entry Medical School, 4i Centre for Interventions in Infection, Inflammation and Immunity, University Hospital Limerick, Limerick, Ireland; ^7^Department of Anatomy, All India Institute of Medical Sciences (AIIMS), New Delhi, India

**Keywords:** development, mesoduodenum, mesocolon, peritoneum, posterior abdominal wall, retroperitoneum, surgical anatomy

## Abstract

**Introduction:** The conventional model of abdominal anatomy described multiple mesenteries. Dissection techniques were based on this. Recent studies demonstrate the mesentery is continuous from duodenojejunal flexure to anorectal junction. Given this, it is important to update dissection techniques related to the mesentery in the cadaveric setting.

**Materials and Methods:** A technique of mesenteric dissection was developed and tested in a cohort of 20 adult human cadavers (12 male and 8 female). As the technique enabled excision of the mesentery as a single unit, it was possible to characterize the anatomy of the *ex vivo* mesentery.

**Results:** The technique developed enabled dissection of an intact and continuous mesentery in all cadavers examined. Examination of the *ex vivo* mesentery demonstrated that a mesoduodenum was present in all cases. The mesentery was continuous from the mesoduodenum to the mesorectum and ended at the level of the anorectal junction.

**Conclusions:** A technique was developed that reproducibly enabled dissection of an intact and continuous mesentery from the duodenum to the anorectal junction. A mesoduodenum was consistently observed and noted to be in continuity with the remainder of the mesentery.

## Introduction

According to the conventional model of abdominal anatomy, the mesentery is defined as a double fold of peritoneum, separated by fatty connective tissue containing vessels and nerves, suspending some parts of the intra-abdominal gut tube from the posterior abdominal wall (1). The name of a specific part is derived from the part of the gut tube that it is attached to e.g., the transverse mesocolon is attached to the transverse colon. “The mesentery” is the term generally applied to the region attached to the jejunum and ileum (1).

The conventional model of mesenteric anatomy describes multiple mesenteries. According to this model, some regions of the intestine have associated mesentery and are “intraperitoneal” whilst others do not, and are described as “retroperitoneal” (1). Recently, this model was challenged with the suggestion that the mesentery is continuous (2–5). Coffey et al. (2) observed, through a series of studies involving intra-operative and radiological anatomy, that the human mesentery is continuous from the duodenojejunal junction to rectum (2–5). Their data suggest that the concept that regions of mesentery regress may not be accurate (6–8).

Recently, we also demonstrated mesenteric continuity in the human cadaveric setting (9). This is not new however. Aristotle (fourth century BC) wrote about it: “The mesenterium is united, and in the middle, it is attached to the aorta.” Galen's (second century) and Mondinus de Luzzi's (Thirteenth century) descriptions of mesentery indicate that they may have considered it continuous (10). Further, a review of the renaissance anatomy and art demonstrates that individuals such as Da Vinci (1452), Andreas Vesalius and Jan Steven van Calcar (1543), Bartolomeo Eustachi (1552), Giulio Casserio (1627) interpreted it as continuous (10, 11). In 1879, the Viennese anatomist Toldt described persistence of the mesentery in association with the ascending and descending colon (12). His illustrations suggest that he interpreted the mesentery of the right and left colon as being flattened against and fused to the posterior abdominal wall. He also indicated that a fascia may be present between the ascending and descending regions of mesocolon and the posterior abdominal wall. This has recently been termed Toldt's fascia (11, 13). However, Gray in the first edition of Gray's Anatomy in 1858 (then called “Anatomy, Descriptive and Surgical”) and Treves (14) presented the mesentery as a fragmented entity (14, 15). Treves, based on findings in cadaveric dissection, argued that a mesentery attached to the ascending and descending colon occurs in a minority of cases (14). Treves views have been largely followed since then.

Support for mesenteric continuity is also found in literature on embryological development of the human, and associated congenital abnormalities. Much of this literature was generated by the anatomists of the nineteenth-twentieth century and is thus limited to the classical textbooks on human anatomy (16–19). According to the conventional model of mesenteric embryology, the mesentery first emerges as a continuous sheet-like structure. Regions of this sheet regress or are obliterated and lost during the later stages of mesenteric attachment and fixation (6, 7, 19). During early embryonic development the entire endodermal gut tube (which forms the gastro-intestinal tract) bears a dorsal mesentery. A limited portion also has contiguous ventral mesentery (foregut). The upper region of the dorsal mesentery develops as a cul-de-sac behind the stomach (containing the developing pancreas and spleen) and then extends caudally below the stomach. Three events between 6th to 10th weeks of intra-uterine life shape the morphological fate of the remaining mesentery; physiological herniation of the midgut through umbilicus, retraction, and in-tandem rotation. Midgut rotation is described as 270 degrees counter-clockwise rotation of the mid gut loop around the root of the superior mesenteric artery (6–8). As a result of midgut rotation, some regions of the mesentery flatten against and become apposed to the posterior abdominal wall. These regions include that which encloses the developing pancreatic buds and contiguous duodenum (with exception of the proximal half of 1st part), that associated with the ascending and descending colon, and rectum. With further development, the posterior mesothelial surface of apposed mesentery is described as fusing with the underlying fascia. As a result of “fusion” these regions of mesentery were interpreted as being lost and the associated region of intestine was described as “retro-peritoneal” (6–8). A remnant of the peritoneal fusion fascia is said to persist in adult on the posterior aspect of the pancreaticoduodenal complex, and beneath the mesocolons opposed to the posterior abdominal wall. On the posterior aspect of head of the pancreas and duodenal loop it is called as Fusion fascia of Treitz (20), and elsewhere as Toldt's fascia (11, 21). Thus, mesentery in the adult intestine is described as restricted in its distribution to the first part of duodenum (derived from ventral mesentery), the duodeno-jejunal to ileocaecal junction—“*the mesentery*,” and transverse and sigmoid colon (6–8).

Several congenital abnormalities support the concept of mesenteric continuity. These include non-rotation and mal-rotation, and can be followed by catastrophic clinical consequences (22). Mesenteric continuity has also been observed in several animal species including coelomic invertebrates, lower vertebrates (23), and in carnivorous reptiles and marsupials (24).

Mesenteric continuity in the adult human is not anticipated by the conventional model of abdominal anatomy. Manuals of human cadaveric dissection are based on the conventional model and now require updating (25, 26). Given this, it is important to develop a reproducible technique for mesenteric cadaveric dissection that preserves mesenteric continuity and thus permits accurate interpretations of the adult topography. The primary aim of the current study was to develop one such technique. Dissection of the mesentery, in a manner that preserves continuity, also enables examination of the *ex vivo* mesentery. Further characterization of the *ex vivo* mesentery formed a secondary aim of the current study.

## Materials and Methods

### Materials

Twenty (12 male and 8 female), donated, adult human cadavers that were embalmed and fixed by infusion of 10% formalin and stored in 10% formalin were used in this study. All the cadavers were examined *in situ* for mesenteric continuity and complete mesenteric specimens were removed from the six cadavers (3 male, and 3 female).

All the cadavers used in this study were donated for medical education and research with due consent of the relatives of the deceased under statutory law and with institutional approval. Any further clearance from the Institutional Ethics Committee was not required.

### Dissection Protocol

A protocol was developed, based on recent reports, enabling dissection of the mesentery in the cadaver. The protocol was applied to all cadavers. The steps of the protocol are as follows:

The anterior abdominal wall was opened and reflected from above downwards as per standard protocol for human cadaveric dissection (25, 26). The greater omentum was reflected upward at its attachment to the greater curvature of stomach. This attachment was divided, enabling exposure of lesser sac. The junction between the pylorus and first part of the duodenum was divided and the stomach reflected cephalad. The transverse mesocolon was separated from underlying structures. This exposed the second and third parts of the duodenum, with the head of the pancreas at the medial border of the duodenum. The head of the pancreas was removed, leaving the body *in situ*. The common bile and main pancreatic ducts were ligated and divided where they entered the second part of duodenum. Jejunal and ileal intestinal loops were reflected toward the left shoulder, thereby exposing the underlying mesentery.

The peritoneal reflection at the lateral aspect of the ascending colon was divided, providing access to the plane formed by the colon and underlying fascia. These were separated, enabling detachment of the colon from the posterior abdominal wall (colofascial separation) (2). The mesocolon was apparent at the medial margin of the colon and the plane formed between it and underlying fascia again disrupted (mesofascial separation) (2). This enabled detachment of the mesocolon. This process was repeated on the right and left side and also at the ileocaecal junction level. Mesofascial separation was continued toward the midline, thereby detaching the entire mesentery as far proximally as the fourth part of the duodenum.

The mesosigmoid was mobilized free from the posterior abdominal wall in a similar manner, the reflection was divided through and the attached region of the mesosigmoid was detached from the underlying fascia toward the midline. Detachment of the mesosigmoid was continued distally under the mesorectum, thereby detaching this from the surrounding musculoskeletal mainframe. This was completed as far distally as was possible, which usually corresponded to the level at which the pelvic floor was encountered. The anorectal junction was then ligated and divided.

At this point the mesentery has been fully detached but remains connected at the major vascular trunks and also at the regions of peritoneal ligamentous connection. These were all then divided and it was then possible to remove the entire mesentery from the abdominal cavity.

In all cases, detailed observations were recorded and photographed.

## Results

### Protocol for Dissection of the Mesentery in the Adult Human Cadaver

A protocol for mesenteric dissection in the cadaver was generated as described above. The protocol was applied to all cadavers and enabled mobilization of the mesentery in each case. The same protocol was applicable irrespective of the sex or age of the cadaver.

### A Continuous Mesentery Was Found Extending From Mesoduodenum to Mesorectum

**Figure 1:**
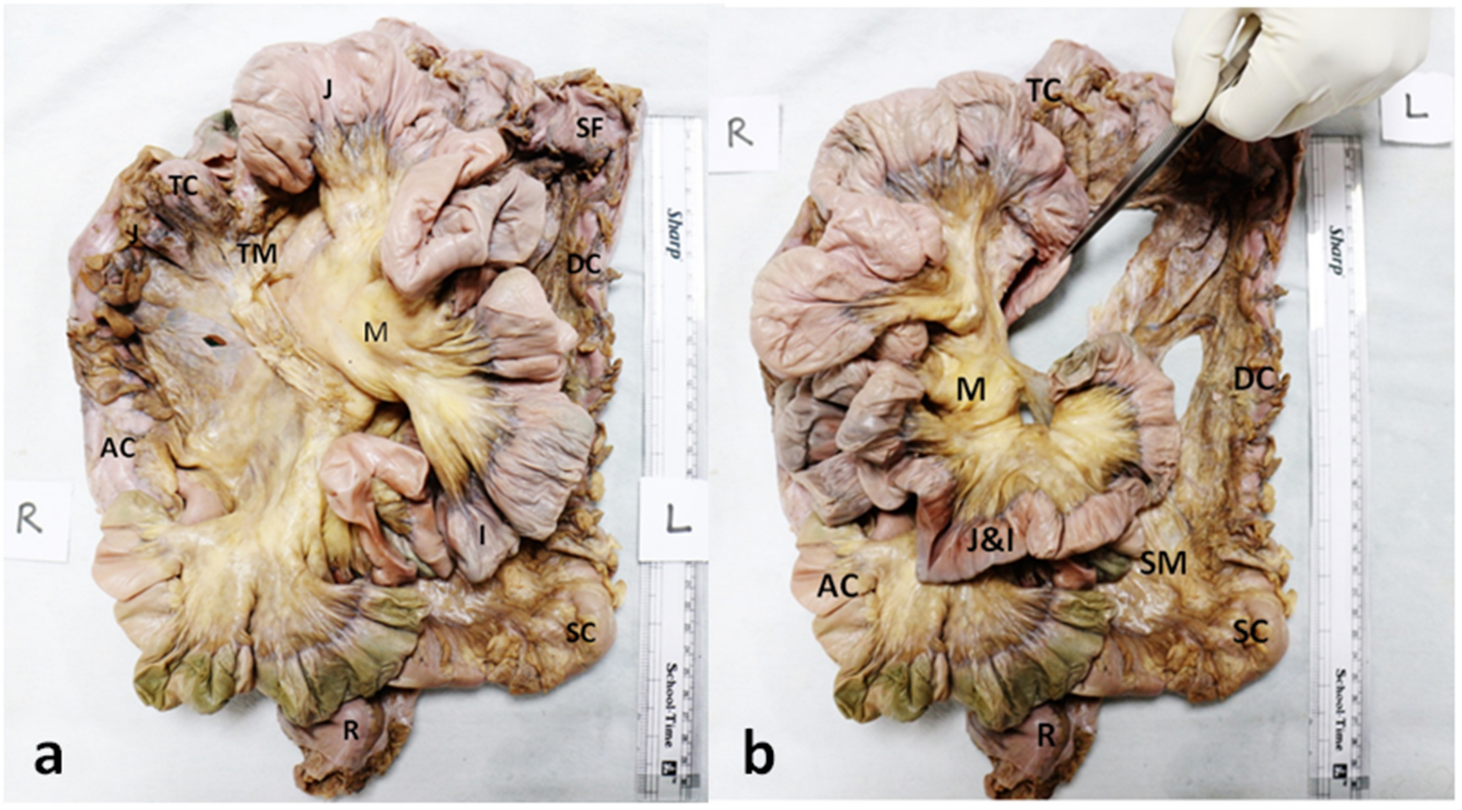
Anterior aspect of composite mesentery. **(a)** Photograph of an intact dissected specimen of the small and large intestines showing the continuity of the mesentery of the ascending (AC) and transverse colons (TC) with the mesentery (M) of jejuno-ileal (J&I) coils (reflected to the left). **(b)** Photograph displaying the continuity of the mesentery of descending (DC), transverse (TC) and sigmoid (SC) colons (J-I coils have been reflected to the left). The forceps are holding up the duodenum (D) (with its mesentery) that can be seen to be passing through a notch created by the folding of the mesentery of jejuno-ileal coils. The sigmoid colon (SC) and its mesentery (SM) are also seen to be continuous with the rectum (R) and its mesentery (thin flap seen lining it to the left).

**Figure 2:**
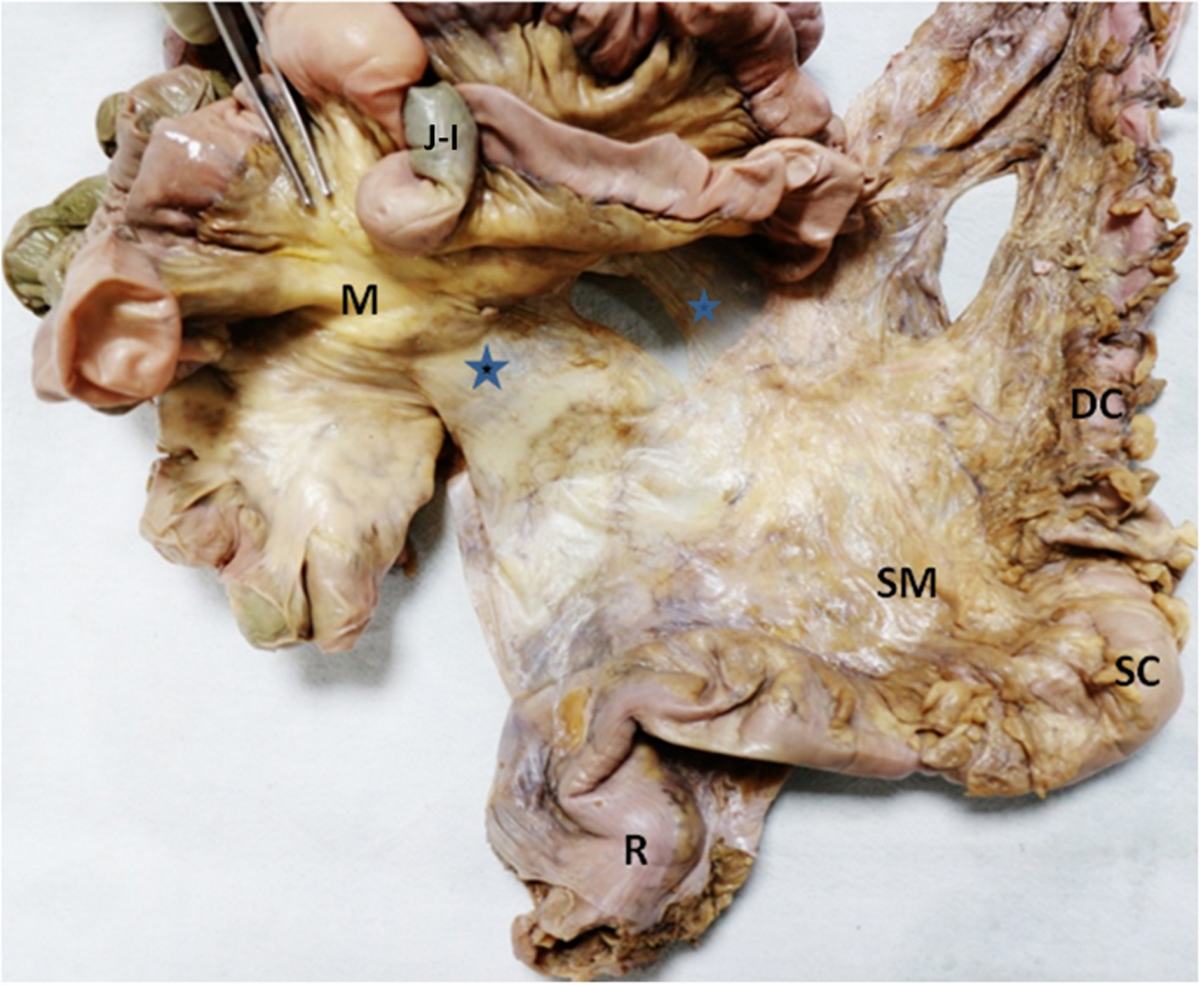
A close-up view of mesenteric continuity of the descending (DC) and sigmoid colons (SC), and rectum (R) from the anterior aspect (after reflecting jejuno-ileal coils laterally). The composite mesentery is seen to continue medially to attach to the left lateral aspect of the mesentery (M) of jejuno-ileal coils (marked with blue stars). The sigmoid colon's mesentery (SM) is seen to be continuous with the mesentery of the rectum.

**Figure 3:**
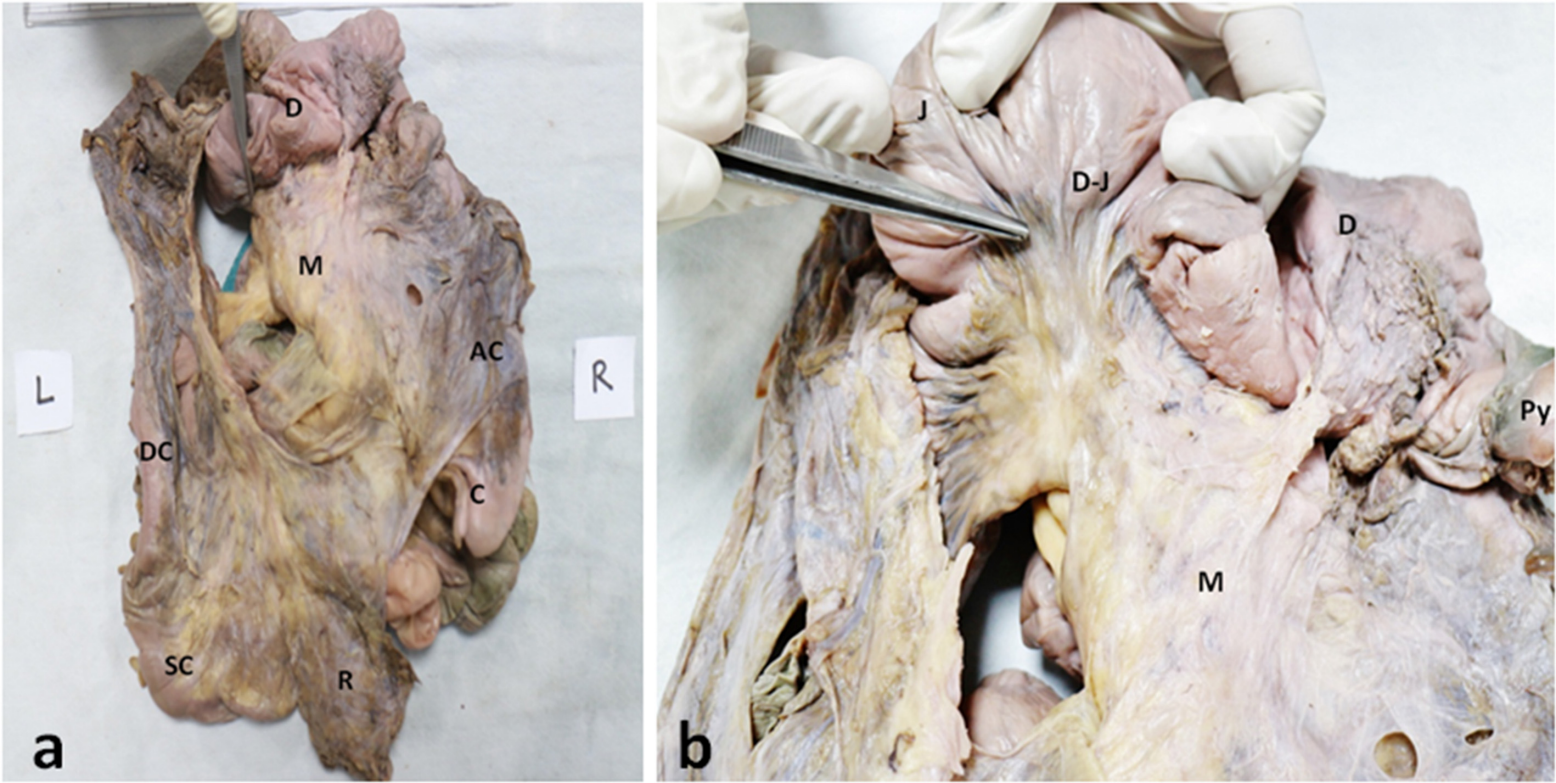
Posterior aspect of composite mesentery. Photographs of **(a)** complete mesenteric specimen displaying the duodenum (D), the mesentery of the jejunum and ileum (M), ascending colon (AC) and caecum (C), descending colon (DC) and sigmoid colon (SC) and the rectum (R). **(b)** In a close-up view, the jejunal mesentery (M) has been pulled back to show its continuity with duodenal mesentery at the duodeno-jejunal junction (DJ); the forceps are pointing to the D-J junction.

**Figure 4:**
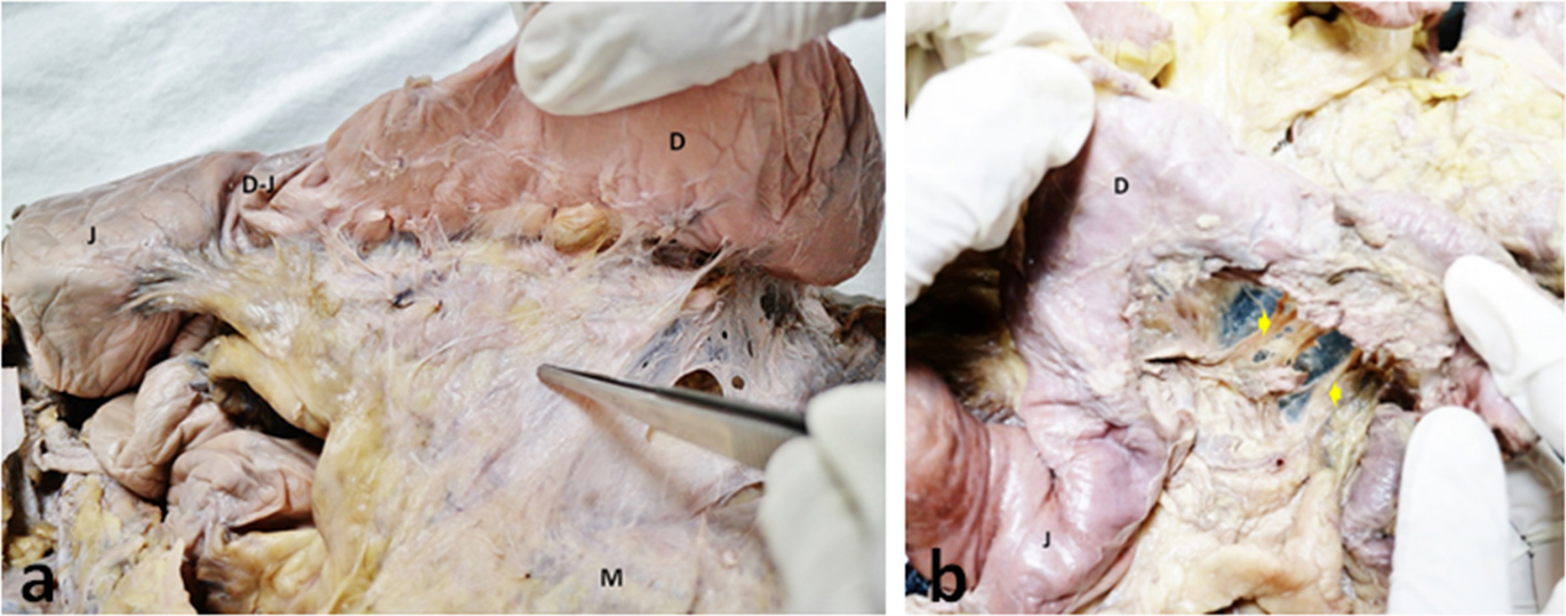
Duodenal mesentery. Photographs of **(a)** a view of duodenal mesentery (indicated by the forceps) also showing the duodenum (D), jejunum (J), duodeno-jejunal junction (DJ), and the mesentery (M). **(b)** Duodenal mesentery dissected to display the neurovascular structures traversing it (as indicated by yellow arrows).

**Figure 5:**
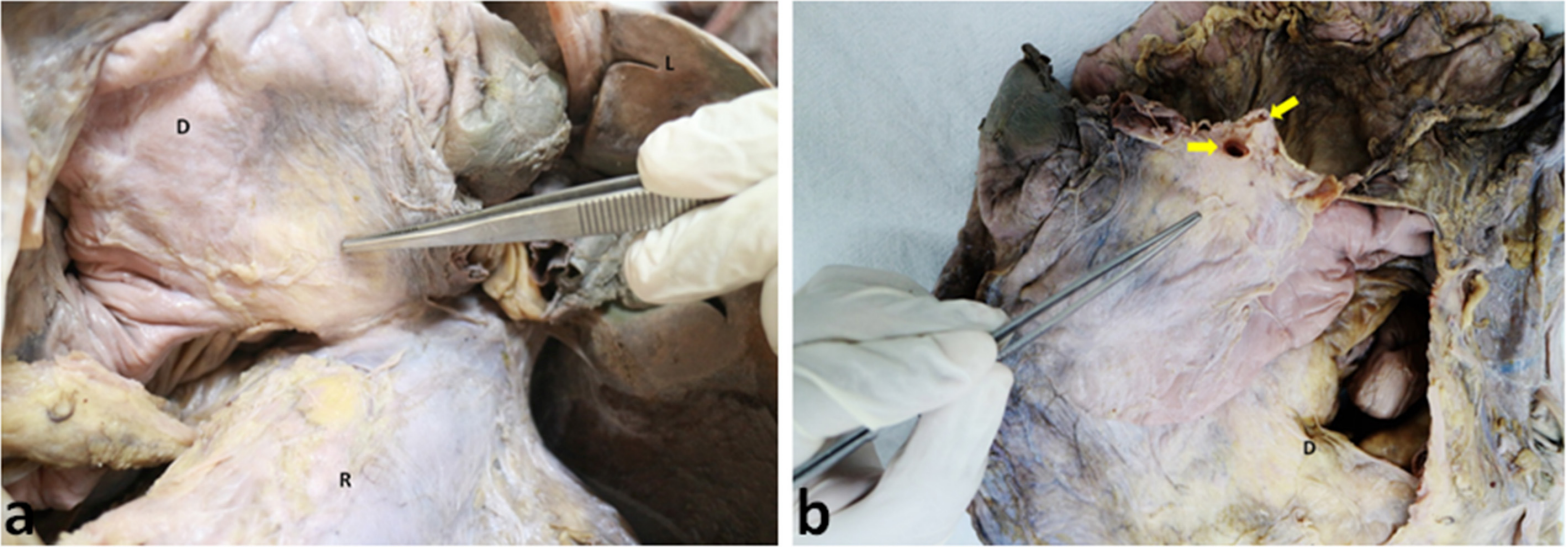
Duodenal mesentery. **(a)** Mesentery of the duodenum (D) (as indicated with forceps) anchored to the root of superior mesenteric vessels. **(b)** Duodenal mesentery has been cut at the root of superior mesenteric vessels (indicated by yellow arrows).

Once the mesentery was dissected a number of observations were made possible. In every case, the mesentery was continuous from the mesoduodenum to the mesorectum (Figures 1–5). This meant the intestine was located at the intestinal margin of the mesentery, from mesoduodenum to mesorectum.

The anterior and posterior aspects of the mesentery were inspected. With exception of the mesoduodenum, all regions of the mesentery were apparent when viewed from front on Figure 1a. The mesoduodenum was only visible by examining the posterior aspect of the mesentery. The mesoduodenum was evident in each cadaver (Figures 1b, 3–5). This was continuous with the small bowel region of the mesentery.

As the mesentery was continuous, distinct anatomical boundaries were not apparent subdividing individual regions. Given this, regions were subdivided based on the region of intestine with which they were continuous. The small intestinal region of mesentery comprised the mesentery associated with the jejunum and ileum. The ileal region of mesentery was continuous above with the transverse mesocolon. The small intestinal region of mesentery was continuous with the mesentery of the ascending colon (the ascending or right mesocolon) (Figures 1a, 2).

The transverse mesocolon was continuous at the splenic flexure, with the mesentery of the left colon (the left mesocolon or descending mesocolon). The latter continued distally as the mesosigmoid which in turn led onto the mesorectum (Figures 1b, 2). The mesentery narrowed at the junction between the mesosigmoid and mesorectum. The mesorectum tapered in width toward the anorectal junction at which level it was negligible (Figure 2).

### The Mesoduodenum

The duodenum and mesoduodenum were located posterior to the composite mesentery of the small intestine, ascending and transverse colons (Figures 1b, 3–5). The small intestinal mesentery, ascending and transverse mesocolon converged centrally toward the root region of the mesentery. In all cases a defect or notch (designated the “mesenteric notch”) was apparent at this level, through which the fourth part of the duodenum emerged and continued as the jejunum (Figure 1b). The notch was obscured from direct visualization on initial examination of the abdomen, and required retraction of jejuno-ileal coils of the small intestine to the right. This notch was not apparent when jejuno-ileal coils were placed back in position. Where the duodenum passed though the notch, a mesenteric twist or spiral was apparent, as the mesoduodenum changed orientation to continue as the mesentery of the jejunum (Figure 4a).

### Mesenteric Vasculature

**Figure 6:**
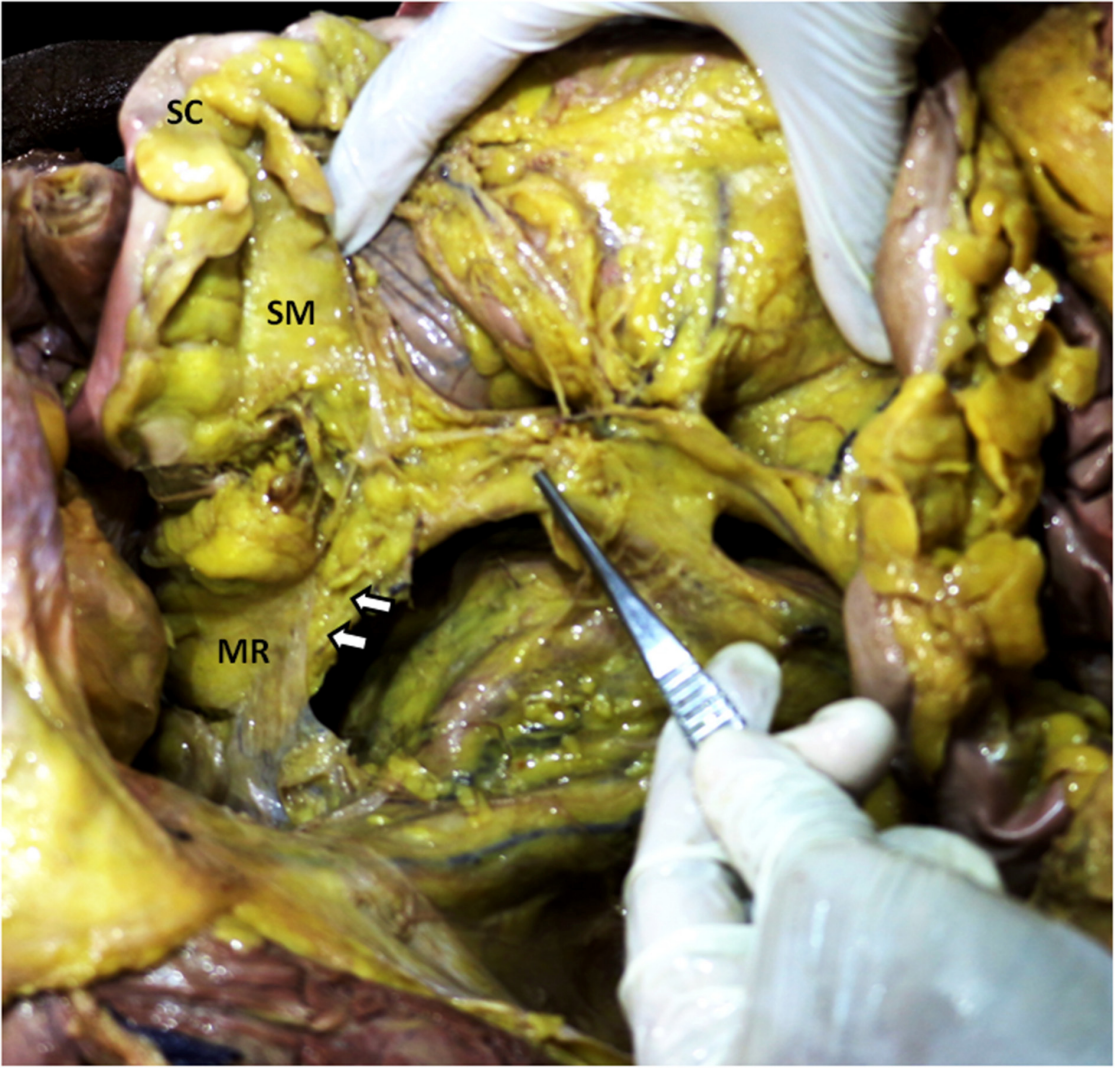
Inferior mesenteric vessels traversing through rectal mesentery. Photograph showing a pair of forceps pointing to the inferior mesenteric vessels. The arrows indicate a fold raised by the inferior mesenteric vessels as they enter the rectal mesentery (MR) after passing through the root of the sigmoid mesentery (SM) attached to the sigmoid colon (SC).

In all cadavers the mobilized mesentery was connected to the posterior abdominal wall at the superior and inferior mesenteric arteries (Figures 5a,b, 6). All branches of these arteries were located within the mesentery. The mesoduodenum had a medial margin, at which the superior mesenteric vessels (vein and artery) were located (Figure 5b). Neurovascular structures passed to and from the duodenum, within the substance of the mesoduodenum (Figure 4b). The regional anatomy of the male and female mesentery overlapped considerably in terms of overall and regional mesenteric topography.

## Discussion

According to surgical dissections (2, 27), the mesentery is continuous and the presence of continuity from duodenojejunal flexure to anorectal junction is now considered as normality. However, few anatomical studies have characterized the mesentery in the cadaveric setting. The present study focused solely on cadaveric specimens and demonstrated mesenteric continuity from mesoduodenum to mesorectum. A protocol was first developed that enabled mesenteric mobilization and excision in the cadaveric setting. The steps of the protocol were repeated in each of the cadavers examined, indicating they may apply to the dissection of human cadavers in general. In dissecting the cadaveric mesentery several observations were enabled. Firstly, the mesentery was found to be continuous from the mesoduodenum to the mesorectum. The entire intestine from duodenum to rectum was located at the intestinal margin of the mesentery, to which it was directly attached. Mesoduodenal, ascending and descending mesocolic regions of mesentery were apparent in all cases.

The present study confirmed the presence of regions of the mesentery associated with both the ascending and descending regions of colon. In the undisturbed state, these were flattened against the posterior abdominal wall (but separate from it). Previous studies have mistakenly interpreted these regions of the mesentery as parietal peritoneum of the posterior abdominal wall.

According to the conventional model of mesenteric anatomy, the root of the mesentery corresponds to the area where the small intestinal mesentery inserts directly into the posterior abdominal wall (1, 28). As per the conventional interpretation, the root extends obliquely across the posterior abdominal wall from the duodenojejunal flexure to the ileocaecal junction. The findings of this study confirm that of Coffey et al. (2), Coffey and O'Leary (3), Culligan et al. (4), and Sehgal and Coffey (5) in showing that the small intestinal mesentery does not insert into the posterior abdominal wall in the manner described in conventional texts, but instead continues laterally to the right as the mesentery of the ascending colon. However, in contrast to the findings of Coffey et al., we found that the small intestinal region of mesentery was also continuous with the mesentery of the descending colon. Based on this it can be argued that there is no true “root” of the mesentery. During embryological development the mesentery is continuous (6–8). This and other recent findings indicate that more proximal regions of the mesentery may also persist in the adult (3, 4). The present findings support this suggestion as a mesoduodenum was apparent in all cadavers examined (9). In all cases, the mesoduodenum was positioned posterior to the main body of the mesentery, a point which may explain its omission in earlier studies. The presence of a mesoduodenum in all cadavers suggests that its presence in the adult human may hold universally, and not just be a geographic-determined anomaly specific to the cohort examined.

Here, we have developed a technique that consistently enabled us dissect an intact and continuous mesentery from the duodenum to the anorectal junction. We found a mesoduodenum in all specimens and it was continuous with the adjacent part of the mesentery.

It is likely that the dissection approach described in this study, and designed specifically to excise an intact and continuous mesentery, will assist students of anatomy, surgery, and radiology in further exploring the mesentery. Classical methods of cadaveric dissection lead the student with the erroneous impression there are multiple individual regions of mesentery (25, 26). The correlation of anatomic, surgical, and radiological imaging of the continuous mesentery is likely to greatly assist the translation of the findings of this study to clinically benefit patients (27).

## Data Availability Statement

The raw data supporting the conclusions of this manuscript will be made available by the authors, without undue reservation, to any qualified researcher.

## Ethics Statement

The patients/participants provided their written informed consent to participate in this study.

## Author Contributions

AK, TJ, and VK conceived and designed the study. AK, MF, HK, VK, TJ, and GR acquired, analyzed, and interpreted the data. AK and TJ wrote the manuscript. JC edited the manuscript. All authors provided approval for the final manuscript.

### Conflict of Interest

The authors declare that the research was conducted in the absence of any commercial or financial relationships that could be construed as a potential conflict of interest.
